# Henoch-Schönlein Purpura in children: not only kidney but also lung

**DOI:** 10.1186/s12969-019-0381-y

**Published:** 2019-11-21

**Authors:** Giada Maria Di Pietro, Massimo Luca Castellazzi, Antonio Mastrangelo, Giovanni Montini, Paola Marchisio, Claudia Tagliabue

**Affiliations:** 1 Fondazione IRCCS Ca’ Granda Ospedale Maggiore Policlinico, Department of Pathophysiology and Transplantation, Pediatric Highly Intensive Care Unit, Università degli Studi di Milano, 20122 Milan, Italy; 2 ASST NORDMILANO, Ospedale di Sesto San Giovanni, Pediatric and Neonatology Unit, Sesto San Giovanni, 20099 Milan, Italy; 3 Fondazione IRCCS Ca’ Granda Ospedale Maggiore Policlinico, Department of Clinical Sciences and Community Health, Pediatric Nephrology and Dialysis Unit, Università degli Studi di Milano, 20122 Milan, Italy; 4 Fondazione IRCCS Ca’ Granda Ospedale Maggiore Policlinico, Pediatric Highly Intensive Care Unit, 20122 Milan, Italy

**Keywords:** Henoch-Schönlein Purpura, IgA Vasculitis, Pulmonary involvement, Diffuse alveolar hemorrhage, Children

## Abstract

**Background:**

Henoch-Schönlein Purpura (HSP) is the most common vasculitis of childhood and affects the small blood vessels. Pulmonary involvement is a rare complication of HSP and diffuse alveolar hemorrhage (DAH) is the most frequent clinical presentation. Little is known about the real incidence of lung involvement during HSP in the pediatric age and about its diagnosis, management and outcome.

**Methods:**

In order to discuss the main clinical findings and the diagnosis and management of lung involvement in children with HSP, we performed a review of the literature of the last 40 years.

**Results:**

We identified 23 pediatric cases of HSP with lung involvement. DAH was the most frequent clinical presentation of the disease. Although it can be identified by chest x-ray (CXR), bronchoalveolar lavage (BAL) is the gold standard for diagnosis. Pulse methylprednisolone is the first-line of therapy in children with DAH. An immunosuppressive regimen consisting of cyclophosphamide or azathioprine plus corticosteroids is required when respiratory failure occurs. Four of the twenty-three patients died, while 18 children had a resolution of the pulmonary involvement.

**Conclusions:**

DAH is a life-threatening complication of HSP. Prompt diagnosis and adequate treatment are essential in order to achieve the best outcome.

## Background

Henoch–Schönlein purpura (HSP), also known as anaphylactoid purpura or, most recently, IgA Vasculitis (IgAV), is the most common leukocytoclastic systemic small-vessel vasculitis in childhood [1]. It is usually characterised by non-thrombocytopenic purpura, with typical purple, non-blanching papules, localised primarily on the lower extremities and buttocks. It can also be associated with abdominal discomfort, joint symptoms such as arthritis or arthralgia of large joints of the lower extremities, and risk of renal involvement [2].

The range of clinical manifestations of HSP is very broad, varying from asymptomatic microscopic hematuria to proteinuria and acute kidney injury (AKI) of various degrees up to rapidly progressive glomerulonephritis with significant risk of chronic renal impairment [3]. Other possible organ involvement includes cerebral vasculitis, ureteral or bladder disease, and scrotal, penile or testicular hemorrhage [4–6].

Piram et al., in a review published in 2013, estimated the annual incidence of HSP in children to be between 3 and 26.7/100,000, demonstrating that it occurs two to 33 times more frequently in infants than in adults [7].

HSP is usually a self-limiting disease with an average duration of 4 weeks; it can have a remitting-relapses course especially within 3 months after initial resolution and rarely, when complicated, it can be fatal [8].

Although the etiology remains unknown, a possible link between genetic predisposition and environmental factors are considered crucial for the pathogenesis of HSP [9, 10].

The diagnosis of HSP is based on clinical findings. The diagnostic criteria for HSP were recently revised by various study groups (EULAR European League Against Rheumatism, PRINTO Pediatric Rheumatology International Trials Organisation and PRES Pediatric Rheumatology European Society), and are listed in Table 1 [11, 12].

**Table 1:** EULAR/PRINTO/PRES diagnostic criteria of Henoch-Schönlein Purpura

Palpable non-thrombocytopenic purpura (mandatory criterion) with lower limb predominance in the presence of at least one of the following four features:	
- Abdominal pain	
- Histopathology showing typical leukocytoclastic vasculitis with predominant IgA deposition or proliferative glomerulonephritis with predominant IgA deposition	
- Arthritis (acute, any joint) or arthralgia	
- Renal involvement (any hematuria and/or proteinuria)	

Pulmonary involvement in HSP is a rare but severe complication and its presence increases the vasculitis morbidity and mortality rates [13–16].

Lung involvement in children with HSP is extremely rare. In particular, sub-clinical lung impairment without respiratory symptoms assessed by pulmonary function tests has been seen during the active phase of the disease, suggesting that the course of the disease involves a malfunction of the alveolar-capillary membrane due to the deposition of immune complexes containing IgA within the vessels of the alveolar septa [17–19].

Chaussen et al. reported a decrease in lung transfer for carbon monoxide (TLCO) in 28 out of 29 patients with HSP without pulmonary symptoms, with normal lung volumes and normal blood gas values, although mild radiological signs of interstitial lung involvement were observed in 18 of 26 patients. In all cases, the TLCO values normalised after complete clinical recovery [20].

TLCO impairment is associated with granular immune complex deposition, neutrophilic interstitial infiltration (acute inflammation), fibrin deposition and consequent fibrinoid necrosis, finally resulting in a thickening of the capillary wall. Nuclear debris may be present in the interstitium, representing leukocytoclasis [21, 22].

This hypothesis is supported by the results of autopsies on patients who died of diffuse alveolar hemorrhage (DAH), which were characterised by pulmonary edema with intra-alveolar hemorrhage, interstitial fibrosis, arterial neutrophilic infiltrate, loss of nuclear staining in the alveolar walls and septa, and capillary wall necrosis. The immunochemistry examination shows granular deposition of IgA along the alveolar septa, suggesting an immune pathogenesis [23, 24].

The absence of IgA on the lung biopsy does not rule out a potential role in the pathogenetic mechanism, due to the rapidity of the immunofluorescence changes, for example on the skin, where IgA disappears just a few days after its deposition [25, 26].

Massive lung involvement such as DAH is rare, especially in the pediatric age. Patients with pulmonary hemorrhage have kidney involvement in up to 95% of cases, more than double the percentage of patients with no pulmonary involvement, sign of the presence of a more severe disease [27, 28].

The aim of this review is to provide a critical appraisal of the medical literature regarding the lung involvement in HSP in children.

## Methods

A research of the literature was performed in PUBMED database, using the terms “Henoch-Schönlein Purpura”, “anaphylactoid purpura”, “Diffuse alveolar hemorrhage”, “Pulmonary Involvement”, “Children”. Only those articles that were reported in English literature and included pediatric patients (0–18 years) with a HSP and lung involvement were included for this narrative review. We found 23 cases between 1979 and 2019, and we described symptoms, diagnostic findings, treatment and outcomes.

## Results

Between 1979 and 2019, there were 23 pediatric case-reports of children with HSP who developed DAH. The main findings of these case-reports are described in Table 2.

**Table 2:** Summary of all reported cases of Henoch-Schönlein Purpura with lung involvement in children

Author, year of publication, nation	Patients (sex, age)	Clinical presentation of HSP	Time between HSP symptoms and pulmonary involvement	Symptoms	Diagnostic findings	Auto-antibody and Kidney Biopsy	Treatment and Outcome
Weiss et al. 1979, USA [23]	M, 8 years	Earache and sore throat (3 days prior to admission), painful swollen joints (2 days prior to admission), skin rash, arthralgia, abdominal pain (1 day prior to admission)	The day of admission to hospital, 2 days after HSP onset	Dyspnea, hemoptysis, tachypnea, nasal flaring, retractions, bilateral basilar rales, anemia	CXR: increased heart size, increased perihilar bronchovascular markings, right pleural effusion. BAL: not performed.	FR and ANA negative. Kidney biopsy not performed.	Oral Steroid and supportive therapy. Not reported mechanical ventilation. Death.
Leatherman et al. 1982, USA [29]	F, 10 years	Skin rash, arthralgia, abdominal pain, nephritis	Not reported	No respiratory symptoms	Lung biopsy: Alveolar hemorrhage, immunofluorescent-negative, with focal chronic alveolar septal inflammation, no vasculitis. BAL: not performed.	C3, C4 normal, Kidney biopsy: granular deposits of IgG, IgM and C3 along glomerular capillary walls.	Oral Steroid + immunosuppressive therapy. Not reported mechanical ventilation. Resolution (after 4 reactivations in 6 months)
Payton et al. 1987, UK [30]	F, 17 years	Skin rash, arthralgia, sore throat and dry cough, proteinuria, hematuria, serum creatinine elevation	2 weeks after clinical manifestation of HSP	Hemoptysis, chest pain, dyspnea, anemia	CXR: bilateral fluffy opacities, consistent with intra-alveolar hemorrhage. BAL: not performed.	Complement screen normal, ANA and anti-GBM negative. Kidney biopsy: patchy tubular loss, no arterial damage, some glomeruli with focal segmental proliferation with foci of necrosis and 4 glomeruli showed crescent. C1, C3, IgA, IgG, fibrin deposits	Oral steroid + immunosuppressive therapy. Resolution.
Olson et al. 1992, USA [10]	F, 14 years	Arthralgia, abdominal pain for 3 months, then skin rash	3 weeks after skin rash	Fatigue, chest pain, cough and rales, hypoxemia, hemoptysis, anemia	CXR: marked fluffy alveolar infiltrates. BAL: Diffuse bleeding.	ANA, ANCA, Anti- GBM negative, C3, C4, IgG, IgA, IgM normal. Kidney biopsy: Focal segmental glomerulonephritis with mild mesangial proliferation; No deposition of IgG, IgM, IgA, C3.	Methylprednisolone iv followed by oral steroid. Red blood cell transfusion. Resolution (only one mild episode of purpura within a few weeks of the pulmonary hemorrhage).
	F, 4½ years	Skin rash, abdominal pain, arthralgia, hematuria	Seven months after onset of HSP but during a fourth discrete episode of reactivation	Fatigue, pallor, cough, chest pain, shortness of breath, hemoptysis, anemia	CXR: reticulo-nodular interstitial pattern. BAL: not performed.	ANA, Anti-GBM negative, C3 decreased, C4, IgG, IgA, IgM normal. Kidney biopsy: Mesangial proliferative glomerulonephrtis with crescenteric lesions (60% glomeruli) No deposition of IgG, IgM, IgA, C3.	Oral steroid + immunosuppressive therapy. Resolution (mild transient recurrence of arthralgia and abdominal pain 16 months after the pulmonary hemorrhage).
	M, 15 years	Skin rash, arthralgia, myalgia, abdominal pain, blood in stools, sore throat, hypertension	5 days after HSP onset	Acute respiratory distress and anemia	CXR: extensive interstitial and alveolar changes. BAL: not performed.	C3, C4 normal.	Oral steroid then methylprednisolone iv. Mechanical ventilation. Red blood cell transfusion. Death.
	M, 16 years	Skin rash, arthralgia, abdominal pain, acute abdomen, hypertension, ARF, weakness and hyperreflexia, fever	3 weeks after onset of HSP	Dyspnea, cough, anemia	CXR: cardiomegaly, pleural effusion, increased pulmonary vascularity CT-scan of lungs: interstitial infiltrates. BAL: not performed.	ANA, ANCA, Anti- GBM negative, C3, C4 normal, IgG, IgA increased, IgM decreased. Kidney biopsy: Diffuse proliferative glomerulonephritis with focal segmental necrosis. Deposition of IgG, IgA, IgM, C3, fibrinogen.	Methylprednisolone iv followed by oral steroid + immunosuppressive therapy. Red blood cell transfusion. Resolution.
Wright et al. 1994, USA [26]	F, 14 years	Skin rash, arthralgia, abdominal pain, melena, hematuria, proteinuria, serum creatinine elevation, ARF	3 weeks after onset of HSP	Hemoptysis, decrease in hematocrit	CXR: bilateral pulmonary infiltrates consistent with hemorrhage Lung biopsy: intra-alveolar hemorrhage with transmural neutrophilic infiltration of small arterioles, capillaries and postcapillary venules, intravascular and intra-alveolar fibrin, IgM, C3 and fibrinogen deposits were present in the small vessels and alveolar capillaries BAL: not performed.	ANCA, ANA, anti-GBM, FR, IgA fibronectin complexes negative. Kidney biopsy: diffuse proliferative endocapillary glomerulonephritis with segmental necrosis and intracapillary fibrin deposition. IgA, IgM, C3 deposits.	Methylprednisolone iv followed by oral steroid. Mechanical ventilation, Peritoneal dialysis. Resolution.
Carter et al. 1996, USA [31]	M, 15 years	Skin rash, abdominal pain, arthralgia, blood in stools, hematuria and proteinuria, hypertension, serum creatinine elevation, fever, sore throat	2 days after admission, 4 days after onset of HSP	Pleural effusion (which was drained), dyspnea, acute respiratory failure, anemia	CXR: infiltrates in the left upper and lower lung fields. BAL: not performed.	C3 normal, C4 low, ANA negative. Kidney biopsy not performed.	Methylprednisolone iv followed by oral steroid. Mechanical ventilation. Red blood cell transfusion. Resolution.
Paller et al. 1997, USA [8]	M, 12 years	Epistaxis, arthralgia, myalgia, skin rash, hypertension, nephritis with ARF	5 days after hospital admission, 12 days after onset of HSP	Tachypnea, cough, hemoptysis	CXR: bilateral fluffy alveolar infiltrates. BAL: not performed.	ANA, ANCA negative, C3, C4, cryoglobulins, IgG, IgA, IgM normal. Kidney biopsy: necrotizing glomerulonephritis.	Methylprednisolone iv followed by oral steroid + immunosuppressive therapy. Mechanical Ventilation. Resolution.
	F, 21 month	Skin rash in the previous months during upper and lower respiratory infections	One week after the onset of an HSP flare (cough, irritability, lethargy)	Pallor and dyspnea, hematuria, anemia	CXR: enlarged heart, extensive bilateral lung infiltrates. BAL: not performed.	Not performed.	Mechanical ventilation. Death.
Vats et al. 1999, USA [32]	M, 7 years	Abdominal pain, vomiting, fever, and 4 days later: skin rash, edema, bloody diarrhea, nephritis (proteinuria and hematuria, serum creatinine elevation)	3 days after the onset of HSP symptoms, 7 days after hospital admission	Respiratory distress (tachypnea, intercostal reactions, chest rales, hypoxemia), anemia	CXR: bilateral pleural effusion with basal pulmonary infiltrates. BAL: not performed.	ANA, ANCA negative, C3 and C4 decreased, IgG decreased, IgA normal. Kidney biopsy: focal and segmental endothelial and mesangial proliferation with no crescents; IgA, C3, and properdin in the mesangium and along the glomerular basement membrane in a peripheral lobular distribution.	Methylprednisolone iv followed by oral steroid. Mechanical Ventilation. Resolution.
Besbas et al. 2001, Turkey [33]	M, 6 years	Skin rash, ARF (edema, hypertension, oliguria and proteinuria)	9 days after skin rash, 2 days after ARF	Dyspnea and anemia	CXR: diffuse pulmonary infiltrates. BAL: not performed.	C3, C4, IgA, IgG, ANA, anti-dsDNA, ANCA, anti-GBM, anti-cardiolipin negative. Kidney biopsy: diffuse proliferation, segmental scars and epithelial-fibroepitelial crescent formation, IgA and mild C3 and IgM staining.	Methylprednisolone iv followed by oral steroid + immunosuppressive therapy. Hemodialysis and Mechanical ventilation. Resolution.
Al Harbi et al. 2002, Kingdom of Saudi Arabia [34]	F, 9 years	Skin rash, knee joint pain, 2 days later abdominal pain and bloody stools and then nephritis with ARF (anuria, edema and hypertension)	10 days after onset of HSP	Respiratory distress, bleeding	CXR: moderate bilateral lung infiltrate and opacities. BAL: not performed.	C3 1.17 g/l, C4 0.22 g/l, ANA 1:320, anti-dsDNA e ANCA negative. Kidney biopsy not performed.	Oral steroid followed by methylprednisolone + immunosuppressive therapy. Then oral steroid + immunosuppressive therapy. Hemodialysis, Mechanical ventilation. Resolution.
Kalyoncu et al. 2006, Turkey [25]	M, 13 years	A one-week history of skin rash and arthralgia followed by abdominal pain and severe mitral insufficiency leading to hospital admission, ARF	3 weeks after the onset of skin rash and arthralgia, 2 weeks after hospital admission	Respiratory distress, severe anemia followed by anuria	CXR: bilaterally diffuse pulmonary opacities. BAL: not performed.	C3 and C4 normal, ANA, anti-dsDNA, LE cell and other rheumatologic markers negative. Kidney biopsy not performed.	Oral steroid, then methylprednisolone iv + immunosuppressive therapy. Mechanical ventilation, Hemodialysis, Red blood cell transfusion. Death.
Matsubayashi et al. 2008 [15]	M, 6 years	Skin rash, 3 days later arthralgia, abdominal pain, blood in stools, microhematuria and then proteinuria	17 days after HSP flare	Dyspnea, polypnea, anemia	CXR: consolidation in the right lung fields. Lung CT scan: diffuse consolidation and patchy opacities mainly in the right lung without interstitial thickening and fibrosis. BAL: not performed.	ANA, ANCA, anti-GBM undetectable. Kidney biopsy: mesangial proliferative glomerulonephritis with crescent formation and IgA deposition in the mesangium.	Oral steroid then methylprednisolone iv + immunosuppressive therapy, then oral steroid + mizoribine. Red blood cell transfusion. Resolution.
Boyer et al. 2011, USA [35]	F, 9 years	Skin rash, arthralgia, abdominal pain, nephritis for 10 days, followed by clinical improvement and the same week, by a flare characterised by rash, arthralgia, abdominal pain, ARF, seizures	20 days after HSP onset	Dyspnea, hypoxemia, and respiratory failure	CXR: diffuse patchy infiltrates. BAL: Gross pulmonary hemorrhage with fresh blood and hemosiderin-laden macrophages	Kidney biopsy: sclerosing lesions and abundant IgA deposits in more than half of the glomeruly. No data about Auto-Antibody.	Methylprednisolone iv followed by oral steroid, then methylprednisolone iv + immunosuppressive therapy. Plasmapheresis, Intravenous gamma globulin, Mechanical ventilation. Resolution (progressive decrease in renal function and 14 months after HSP onset, successful kidney transplant)
Chen et al. 2011, Taiwan [14]	F, 11 years	Skin rash, abdominal pain, vomiting for 3 days then hematuria and nephrotic proteinuria, ARF	3 days after hospital admission, 6 days after HSP onset	Cough, general malaise, anemia	CXR: bilateral infiltration and consolidation Lung CT scan: multiple patches with ground-glass appearance. BAL: Macrophages with hemosiderin-laden particles and numerous red blood cells	C3 167 mg/dl, C4 43.2 mg/dl, ANA 1:40, anti-dsDNA, ENA, anti-GBM, anti-cardiolipin, cryoglobulin, cANCA negative, pANCA positive. Kidney biopsy: crescentic glomerulonephritis with diffuse mesangial proliferation and focal necrosis, IgA deposition.	Oral steroid then methylprednisolone iv followed by oral steroid + immunosuppressive therapy, plasma exchange Red blood cell transfusion. Resolution.
Ren et al. 2013, China [36]	F, 11 years	Skin rash for 2 weeks and urine test abnormality for 3 days, hospitalised with skin rash, joint pain, abdominal pain, and 10 days later nephritis	Not reported	No signs	CXR and Lung CT scan compatible with hemorrhage. BAL: not performed.	C3, C4 normal. ANA, ENA negative. Kidney biopsy: mesenteric and endothelial cell proliferation and lobulation; three segments mild mesenteric proliferation and focal segmental hypertrophy of podocytes, and two segments presented endothelial cell swelling. Deposit of IgA and C3.	Oral steroid. Resolution.
Ngobia et al. 2014, USA [27]	M, 18 years	Skin rash, arthralgia, abdominal pain, dark urine for 5 days, followed by hospital admission with finding of hematuria and proteinuria, treated with prednisolone and discharged after 4 days.	15 days after HSP onset, 6 days after discharge	Shortness of breath, hemoptysis, epistaxis and anemia, associated with worsening of HSP symptoms and hematuria, nephrotic proteinuria and serum creatinine elevation	CXR: right lower lobe opacity Lung CT scan: mixed ground glass appearance Lung Function Tests: normal. BAL: Low inflammatory profile and 30% hemosiderin-laden macrophages	C3, C4 normal. ANA, anti-GBM, ANCA negative. Kidney biopsy: necrotizing segmental glomerulonephritis with strong positive IgA and C3 staining.	Oral steroid, followed by methylprednisolone iv, then oral steroid + immunosuppressive therapy. Resolution.
Aeschlimann et al. 2017, Canada [37]	M, 26 months	Skin rash, arthralgia, swelling, microhematuria and then proteinuria with hypertension	A few days after onset of HSP	Tachypnea, shortness of breath, severe respiratory distress, anemia	CXR and CT scan of the lungs showed extensive bilateral patchy opacities. BAL: Diffusely blood-tinged mucus, no hemosiderin-laden macrophages	Normal C3, ANA, ANCA, Anti-GBM negative. Kidney biopsy: involvement of 10% of 23 glomeruli with segmental granular staining of mesangial regions with some capillary loops for IgG, IgA and C3 (IgAV nephritis).	Methylprednisolone iv then oral steroid + immunosuppressive therapy. Mechanical ventilation Red blood cell transfusion. Resolution.
James et al. 2017, USA [38]	F, 10 years	Skin rash, arthralgia, fever, dry cough and then severe mitral regurgitation/ diastolic dysfunction/ increase in size of left atrium	10 days after HSP onset	Shortness of breath, tachypnea, anemia	CXR: patchy bibasilar opacities Lung CT scan: bibasilar airspace disease. BAL: not performed.	Kidney biopsy not performed.	Methylprednisolone iv then oral steroid. Mechanical ventilation, Intravenous gamma globulin. Resolution (mild to moderate mitral regurgitation persisted after 1 year).
Clarke et al. 2018, UK [39]	M, 5 years	Purpuric rash, swollen extremities, arthralgia, fever, abdominal pain and rectal bleeding, proteinuria and hematuria (through to deterioration in renal function)	3 weeks after HSP onset	Respiratory deterioration, anemia	CXR: bilateral focal abnormalities. BAL: not performed.	C3, C4, ANA, ANCA, anti-dsDNA negative. Kidney biopsy: diffuse proliferative glomerulonephritis with strong deposition of IgA.	Methylprednisolone iv, then immunosuppressive therapy + plasmapheresis followed by oral steroid and enalapril. Mechanical ventilation, Red blood cell transfusion. Resolution.

*ANA* Anti-nuclear antibody, *ANCA* Antineutrophil cytoplasmic antibody, *ARF* Acute Renal Failure, *anti-GBM* Anti-glomerular basement membrane antibody, *anti-dsDNA* Anti-double stranded DNA, *ENA* anti-extractable nuclear antigen, *LE cell* Lupus erythematosus cell, *FR* Rheumatoid factor

The average age at the onset of HSP with pulmonary involvement was 10.2 years, with little difference between males (12 cases) and females (11 cases).

Almost all children with pulmonary hemorrhage had renal involvement, ranging from hematuria often associated with proteinuria, to nephritis or nephrotic proteinuria through to AKI [8, 10, 14, 15, 25–27, 29–37, 39]. The co-occurrence of rapidly progressive glomerulonephritis and DAH is also known as “pulmonary renal syndrome” and it is primarily related to an autoimmune etiology. However, Gd-IgA1 pathway is probably involved, due to the presence of similar manifestation in other IgA related disorders, such as IgA nephropathy (IgAN), ANCA-associated vasculitis overlap syndrome, IgA-dominant post-infectious glomerulonephritis, IgA-variant Goodpasture’s syndrome and IgA-producing monoclonal gammopathy [40–42].

Twelve of twenty-three children were screened for ANCA: 11 were negative and one was c-ANCA negative but p-ANCA positive (this patient had IgA deposition in kidney biopsy but the possibility of concurrent ANCA-associated vasculitis could not be excluded at all). Eleven of twenty-three children were not screened for ANCA: six of them did not perform kidney biopsy, three had IgA deposition in renal biopsy, two had other glomerular pattern with different deposits in biopsy. Almost all children had a normal complement screening and negative auto-antibody research, so there are no markers which can predict a more severe course of the disease [8, 10, 14, 15, 23, 25–27, 29–34, 36–39].

In the majority of the cases, the pulmonary symptoms included cough, hemoptysis, epistaxis, dyspnea, tachypnea, chest pain and shortness of breath through to acute respiratory failure [8, 10, 14, 15, 23, 25–27, 30–35, 37–39]. However, pulmonary hemorrhage should be considered also in HSP patients with no respiratory symptoms or with common pulmonary manifestations. Two of twenty-three patients had no respiratory symptoms, but only radiological imaging findings suggestive of DAH [29, 36]. So most but not all children with pulmonary vasculitis have respiratory symptoms, in some anemia or incidental radiological findings bring it to attention.

The time between the onset of the disease and the occurrence of the respiratory symptoms ranged from 2 days to 3 weeks, with a mean of 12 days [8, 10, 14, 15, 23, 25–27, 30–35, 37, 38], except for 2 cases in which the timing was not reported due to the absence of these symptoms [29, 36].

The chest x-ray (CXR) was the first diagnostic step for DAH, and in most cases showed a pattern of diffuse bilateral pulmonary infiltrates.

In some cases, due to a misinterpretation of the CXR findings, patients were treated with antibiotic therapy due to a suspicion of a lobar pneumonia instead of DAH [10, 27, 37]. For this reason, bronchoalveolar lavage (BAL) should be performed to confirm diagnosis and start treatment promptly. Five of twenty-three children had BAL performed; the typical findings ranged from the evidence of blood in the alveolar space in the acute phase, to the presence of hemosiderin-laden macrophages in chronic hemorrhage [14, 27, 35, 37, 43].

Eighteen of twenty-three children (78%) had anemia and decreased hematocrit values due to pulmonary hemorrhage [8, 10, 14, 15, 23, 25–27, 30–33, 37, 38], and nine (39%) needed red blood cell transfusion [10, 14, 15, 25, 31, 37].

Thirteen children (56%) required mechanical ventilation, 12 of whom for respiratory distress, and remaining patient for massive pulmonary hemorrhage [8, 10, 25, 26, 31–35, 37, 38]. The need for intubation was not reported for two patients (8%) [23, 29].

The two patients without respiratory symptoms achieved DAH resolution, in one case with oral prednisone therapy alone and in the other with oral prednisone plus azathioprine [29, 36]. Four children with DAH died despite treatment [8, 10, 23, 25]. In the remaining 17 surviving patients (73%), at the time of pulmonary involvement, nine had required no therapy, three were being treated with oral prednisone and five with intravenous pulse methylprednisolone (due to the worsening of the kidney disease or to the massive lung involvement on the computed tomography scan).

Of the nine patients who had not been prescribed any medication, five of them needed mechanical ventilation and all of them achieved resolution with pulse methylprednisolone or with steroid treatment plus immunosuppressive therapy [10, 30–32, 36–38].

The three patients treated with oral prednisone did not need intubation and all survived after receiving pulse methylprednisolone with or without another immunosuppressive drug (cyclophosphamide) [14, 15, 27].

Of the five patients who were administered pulse methylprednisolone, all of them needed mechanical ventilation, three of them continued with pulse methylprednisolone, and for the other two cyclophosphamide was added to the intravenous steroid [8, 26, 33–35].

Before the onset of respiratory symptoms, 12 of the 17 children were not taking any medication or were on oral prednisone (2 mg/kg/day), which could suggest that oral steroid (and not intravenous steroid) seems not to prevent pulmonary involvement. However, those patients on oral steroid therapy did not need mechanical ventilation. The absence of medication increased the risk of acute respiratory failure, so steroid therapy was recommended to avoid intubation and to reduce the mortality rate [14, 15, 27].

No standard therapy was established for patients with HSP with pulmonary involvement; however, the first choice treatment was high-dose intravenous pulse methylprednisolone, with or without immunosuppressive therapy. Oral steroid therapy was ineffective for the prevention of lung involvement, considering that most patients were on oral steroids before the onset of DAH, although it did reduce the risk of acute respiratory failure [14, 19, 36].

Four children died, the lung biopsy of three of them were characterised by atelectasis, pulmonary edema with intra-alveolar haemorrhage, interstitial fibrosis and diffuse iron deposition [8, 10, 23, 25]. Among the 19 surviving patients, 16 had a resolution after the first episode, while three had a recurrent disease: the first repeated arthralgia and abdominal pains, the second a single mild episode of purpura and the third multiple reactivations [8, 10, 14, 15, 26, 27, 29–39].

## Discussion and conclusion

HSP is one of the most common small-vessel vasculitis in children. It is generally a self-limiting disease in which the most feared complication is renal involvement.

Although rare, lung involvement in patients with HSP could be asymptomatic and should be investigated in order to diagnose potential lung involvement, due to a disease progression to a more severe clinical presentation, such as DAH. TLCO could be performed early in patients with HSP to evaluate the presence of pulmonary involvement even in the absence of symptoms, in order to perform more investigations and to begin the appropriate supportive treatment immediately. Indeed the carbon monoxide diffusing capacity tests is lower in children with HSP during the acute phase of the disease and normal during resolution of the disease.

In patients with DAH, the most common clinical findings are cough, hemoptysis, epistaxis, dyspnea, tachypnea, chest pain and shortness of breath through to acute respiratory failure that could require intubation and mechanical ventilation. Limited data are available for the prevention and treatment of lung involvement in HSP. It was observed that oral corticosteroids are not sufficient for the prevention of DAH, but they could reduce the need for mechanical ventilation. No guidelines regarding the treatment of DAH are so far available. Because of the disease progression and the common associated renal involvement, we suggest to treat every pulmonary complication with intravenous methylprednisolone, avoiding to wait an eventually life threatening complication as DAH. However, the best treatment for lung involvement in HSP is still a matter of debate and needs further researches.

Immunosuppressive treatment (azathioprine or cyclophosphamide) is indicated in presence of respiratory failure.

The limits of our article are that it is a narrative review and it is based almost on case reports or small case series. So, further multicentre studies on lung involvement in HSP are essential in order to define a diagnostic protocol, the best therapeutic strategies and their duration and the long-term outcomes.

## Data Availability

Not applicable.

## Abbreviations

AKI: Acute kidney injury
BAL: Bronchoalveolar lavage
CXR: Chest x-ray
DAH: Diffuse alveolar hemorrhage
Gd-IgA1: O-galactosylated-IgA1
HSP: Henoch–Schönlein purpura
IgAV: IgA Vasculitis
TLCO: Lung transfer for carbon monoxide
